# Predictors and Correlates of Perceived Cognitive Decline in Retired Professional Rugby League Players

**DOI:** 10.3389/fneur.2021.676762

**Published:** 2021-10-11

**Authors:** Ryan Van Patten, Grant L. Iverson, Douglas P. Terry, Christopher R. Levi, Andrew J. Gardner

**Affiliations:** ^1^Department of Physical Medicine and Rehabilitation, Harvard Medical School, Boston, MA, United States; ^2^Spaulding Rehabilitation Hospital, Charlestown, MA, United States; ^3^Spaulding Research Institute, Charlestown, MA, United States; ^4^MassGeneral Hospital for Children Sports Concussion Program, Boston, MA, United States; ^5^Home Base, A Red Sox Foundation and Massachusetts General Hospital Program, Charlestown, MA, United States; ^6^Sydney Partnership for Health, Education, Research and Enterprise (SPHERE), Liverpool, NSW, Australia; ^7^School of Medicine, University of New South Wales, Randwick, NSW, Australia; ^8^Priority Research Centre for Stroke and Brain Injury, School of Medicine and Public Health, University of Newcastle, Callaghan, NSW, Australia; ^9^Hunter New England Local Health District Sports Concussion Program, Waratah, NSW, Australia

**Keywords:** retired athletes, sports, brain injury, cognition, subjective cognitive decline, mental health

## Abstract

**Objective:** Rugby league is an international full-contact sport, with frequent concussive injuries. Participation in other full-contact sports such as American football has been considered to be a risk factor for neuropsychiatric sequelae later-in-life, but little research has addressed the mental and cognitive health of retired professional rugby league players. We examined predictors and correlates of perceived (self-reported) cognitive decline in retired National Rugby League (NRL) players.

**Methods:** Participants were 133 retired male elite level rugby league players in Australia. Participants completed clinical interviews, neuropsychological testing, and self-report measures. The Informant Questionnaire on Cognitive Decline in the Elderly, self-report (IQCODE-Self), measured perceived cognitive decline.

**Results:** The median age of the sample was 55.0 (M = 53.1, SD = 13.9, range = 30–89) and the median years of education completed was 12.0 (M = 11.9, SD = 2.6, range = 7–18). The retired players reported a median of 15.0 total lifetime concussions (M = 28.0, SD = 36.6, range = 0–200). The mean IQCODE-Self score was 3.2 (SD = 0.5; Range = 1.3–5.0); 10/133 (7.5%) and 38/133 (28.6%) scored above conservative and liberal cutoffs for cognitive decline on the IQCODE-Self, respectively. Perceived cognitive decline was positively correlated with current depressive symptoms, negatively correlated with years of professional sport exposure and resilience, and unrelated to objective cognition and number of self-reported concussions. A multiple regression model with perceived cognitive decline regressed on age, concussion history, professional rugby league exposure, depression, resilience, objective cognitive functioning, daytime sleepiness, and pain severity showed depression as the only significant predictor.

**Conclusion:** This is the first large study examining subjectively experienced cognitive decline in retired professional rugby league players. Similar to studies from the general population and specialty clinics, no relationship was found between objective cognitive test performance and perceived cognitive decline. Depressive symptoms emerged as the strongest predictor of perceived cognitive decline, suggesting that subjective reports of worsening cognition in retired elite rugby league players might reflect psychological distress rather than current cognitive impairment.

## Introduction

The extent to which repetitive neurotrauma from earlier in life participation in contact and collision sports is related to later-in-life cognitive decline and impairment is not well-understood (1, 2). Most of the research published to date has been conducted in former amateur and professional American football players (2, 3), while there are fewer studies in other contact and collision sports. Survey studies have revealed that some former college and professional American football players report cognitive problems many years following their retirement from football, but the majority rate their functioning as broadly normal (4, 5). Those with a history of multiple concussions are more likely than those without such a history to report later-in-life cognitive problems (4, 6, 7). Two recent review papers suggest that some former American football players perform more poorly on neuropsychological testing than controls, but there are several substantive methodological limitations in the literature that limit the conclusions that can be drawn (3, 8). One mortality study found that rates of neurodegenerative diseases listed as an underlying or contributing cause of death in former professional American football players were significantly greater than expected compared to men from the US general population (9).

In one study of middle aged former *high school* American football players, participants did not report worse brain health or cognitive problems compared to those who played other contact sports, non-contact sports, or did not participate in sports during high school (10). Additionally, a large-scale longitudinal study found that older adult men who played high school American football were not more likely to have cognitive impairment (11), and two medical-record linkage studies relating to former high school American football players found no increased risk for later-in-life neurodegenerative diseases compared to men who did not play American football (12, 13).

### Perceived Cognitive Decline in the General Population and People With Traumatic Brain Injuries

Perceived (i.e., self-reported) cognitive decline is common in the general population, with ~11% of people age 45 or older endorsing some degree of perceived cognitive decline based on a large 2015–2016 random-digit-dialing survey conducted in all 50 states (14). Civilians and Veterans with a history of traumatic brain injuries (TBIs) are more prone to perceive cognitive problems, with 154/227 (68%) of patients presenting to Level 1 trauma centers 6 months following mild TBIs (15) and 24,982/30,267 (83%) of OEF/OIF Veterans who presented for a comprehensive TBI workup at their VA following a positive TBI screen (16) reporting subjective cognitive complaints.

Multiple systematic reviews and meta-analyses in the general population have investigated the relationship between perceived and objective cognition in tens of thousands of participants (17–21). Converging results suggest that, although baseline subjective cognitive complaints do predict later cognitive decline and dementia (19–21), the cross-sectional relationship between subjective and objective cognition is small, with about 1% of the variance shared (17, 18). In contrast, depressive symptoms and psychological factors are consistently reported as strong correlates of perceived cognitive decline in both general population studies (17, 21) and in the TBI literature (15, 22–25).

### Perceived Cognitive Decline in Retired Athletes

There is far less research on perceived cognitive decline in retired athletes compared to the general population. Cunningham et al. (8) conducted a systematic review of long-term outcomes in retired athletes and reported that 11 of 14 (78.6%) studies found higher levels of perceived cognitive problems in retired athletes compared to control groups or normative data. However, many of the studies recruited small samples and none of the studies specified perceived cognitive decline as a primary research question. Guskiewicz et al. (6) examined the relationship between prior concussions and later cognition in retired National Football League (NFL) players, and 17.6% (266/1,513) of the retired players with concussion histories reported feeling that the concussions had negatively impacted their cognitive abilities as they aged. In a recent study (5), researchers measured subjective cognition in 3,758 retired NFL players, of whom 1,502 (40.0%) self-reported daily cognitive problems. Consistent with the aforementioned research in the general population, the retired athletes with perceived cognitive problems were more likely than those without such problems to have co-occurring depression and psychological distress (5).

### Purpose and Hypotheses

Rugby league is a full contact collision sport (26) with frequent tackling (27) and a relatively high rate of concussions (28). The purpose of the current study is to examine predictors and correlates of perceived (self-reported) cognitive decline in retired elite level rugby league players. Although there is a large literature on correlates of perceived cognitive decline in the general population, there is a paucity of evidence in retired professional athletes. Moreover, although depression and personality variables are sometimes accounted for in general population research (17, 21), positive psychological characteristics such as resilience, which may serve as a buffer against negative outcomes in athletes (29), are understudied. Finally, chronic pain, sleep, and substance use behavior are related to negative outcomes in former professional athletes (30, 31), including cognition-related quality of life (32).

We hypothesized that greater perceived cognitive decline would be significantly correlated with *greater* (i) lifetime history of concussions, (ii) number of years participating in professional sport, and (iii) current symptoms of depression. We also hypothesized that (iv) greater resilience would be associated with less perceived decline in cognitive functioning. Regarding the association between subjectively experienced cognitive decline and objectively measured cognitive functioning, we (v) predicted that there would be no significant association. We also (vi) hypothesized that current depressive symptoms would be the strongest predictor of perceived cognitive decline in a multivariable model. Finally, we (vii) explored demographic, concussion-related, and mental/physical health predictors of objective cognitive and memory performance.

## Methods

### Participants

Participants were retired male elite level rugby league players who competed at the first grade level in the New South Wales Rugby League (NSWRL, 1908–1994), Australian Rugby League (1995–1997), Super League (1997), and/or National Rugby League (1998–present). Two methods of recruitment were used. First, “old boys” club (alumni) networks distributed study information to their members. Second, direct and indirect referrals were received from two sources: (i) the National Rugby League (NRL), and (ii) the Men of League Foundation, who also distributed study information to their members and provided the option to self-refer to the research program. Rarely, the NRL or the Men of League Foundation sought permission from a retired player to pass on their contact details to the research team. If the retired player then expressed interest in participation in the study, the research team would make arrangements to schedule the former player. This method of participant recruitment and selection might result in some retired rugby league players with cognitive dysfunction and/or severe depression being less willing or able to participate in the study. That said, there could also be some degree of recruitment bias in the other direction, whereby those who are the healthiest, highest functioning, and who have busy personal and professional lives, might be less likely to participate. We did attempt to mitigate bias in healthy volunteers by recruiting a demographically representative sample of retired rugby league players.

Study exclusion criteria were a medical history of neurosurgery and any history of a brain tumor requiring radiation treatment. To date, 145 retired players have participated. For this study, we excluded twelve men because they did not complete the primary dependent variable (self-reported cognitive decline). Three of these men were found to have dementia and were unable to complete all of the study procedures. The final study sample size was 133.

### Procedures

The parent study, *The Retired Professional Rugby League Players Brain Health Research Program*, began in 2012 and has been modified multiple times. This study was approved by the Institutional Human Ethics Committee. Currently, the study includes a health survey, an in-person clinical evaluation, neuropsychological testing, multi-modal experimental neuroimaging, blood biomarkers and genetic testing, and brain and spinal cord donation. Participants may opt in or out of any portion of the research procedure. The clinical interview collected demographic data, medical and concussion history, and patient-reported outcome measures. Immediately following the interview, participants completed objective neuropsychological testing. All participants completed the clinical interview and neuropsychological testing with the senior author (AJG), which lasted ~2 h and 15 min. All variables used in the current study were collected during the clinical interview, cognitive testing, and from responses to self-report questionnaires.

### Measures

#### Primary Dependent Measure

The Informant Questionnaire on Cognitive Decline in the Elderly (IQCODE) (33–36) has been modified to include a self-report version (IQCODE-Self) (37). The original instrument included 26 items rated on a 1 (“Much improved”) to 5 (“Much worse”) scale assessing decline in memory, comprehension, decision making, reasoning, and instrumental activities of daily living over the previous 10 years. The individual item scores are averaged to create a total score (range: 1–5). A large population-based sample in Australia showed strong internal consistency reliability (α = 0.95) and one-year test-retest reliability (*r* = 0.75) (34). Later, a 16-item short-form was developed (38); its psychometric properties are strong and comparable to the long form (39, 40). Moreover, there is support for the reliability and validity of the self-report version as well. Jansen et al. (37) administered the IQCODE-Self to a large community sample and reported high internal consistency (α = 0.95), positive correlations with measures of disability in activities of daily living, higher scores in older compared to younger adults, similar scores in men and women, and near-zero correlations with education and occupational attainment. This suggests that the instrument measures one primary construct, that it detects decline in activities of daily living and cognition, and that scores do not vary based on demographic variables such as gender, education, and occupation.

Multiple IQCODE cutoffs have been developed to detect cognitive decline and dementia in older adults, with values ranging from conservative (3.88) to liberal (3.38) (35). In the current study, we administered the 16-item IQCODE-Self as a measure of perceived cognitive decline and examined two cutoff values for identifying cognitive impairment: 3.88 and 3.38.

#### Exposure Variables

Concussion history was based on self-report. During the clinical interview, the definition of concussion from the consensus statement on concussion in sport, 2012 (41), was provided to the participants, and they were permitted to questions to clarify the definition. Next, they reported the number of lifetime sport-related and non-sport-related concussions sustained and these values were added together to calculate the total number of lifetime concussions per participant. Number of years played professionally (i.e., at the elite level), number of games played professionally, and total (lifetime) number of years playing rugby league were also based on self-report, and were verified by online secondary sources that record historical match and player data.

#### Questionnaires and Cognitive Tests

The in-person evaluation included the administration of patient reported outcome measures and neuropsychological tests. The patient reported outcome measures of interest in the current study were as follows: the Depression, Anxiety, Stress Scale 21-item (DASS-21) (42), the Connor-Davidson Resilience Scale (CD-RISC) (43), the Epworth Sleepiness Scale (ESS) (44), the Alcohol Use Disorders Identification Test (AUDIT) (45), and the Brief Pain Inventory (BPI) (46).

Cognitive tests measuring attention, processing speed, learning and memory, and executive functions were administered as part of the cognitive test battery. The Advanced Clinical Solutions (ACS) Test of Premorbid Functioning (ToPF) (47) was also administered to establish an estimated level of intelligence for each participant. Given the large number of variables generated by a cognitive test battery, leading to alpha inflation, as well as the diffuse cognitive effects of head injuries, we examined cognition using two composite variables: an overall cognitive composite score and a memory composite score. Each participant's overall cognitive composite was based on 12 test scores; Mitrushina et al. (48) meta-norms converted raw data to standard scores for the Rey Auditory Verbal Learning Test (RAVLT) Trials 1-5 (49), Rey Complex Figure Test (RCFT) Immediate Recall and Delayed Recall (50), Trail Making Test (TMT) A and B (51), the Controlled Oral Word Association Test (COWAT) FAS and Animal Fluency (52), and Stroop condition 3 – Inhibition (53). The Wechsler Adult Intelligence Scale, 4th Edition (WAIS-IV) (54). Australian and New Zealand norms converted the raw data to standard scores for Digit Span (Backwards and Sequencing, separately), Symbol Search, and Coding. Each of the twelve age-adjusted cognitive scores for each participant were converted to T scores (M = 50, SD = 10) if they were not already in that distribution. The twelve scores were averaged to generate a cognitive composite score. Additionally, RAVLT Trials 1-5, RAVLT Short Delay Recall, RCFT Immediate Recall, and RCFT Delayed Recall were averaged to create a memory composite score.

#### Statistical Analyses

Descriptive statistics were calculated for all variables used in the study. Due to non-normality in at least one variable involved in each statistical model, non-parametric models are presented. Spearman correlations were conducted between the IQCODE-Self score and each correlate of interest: DASS depression raw score, CD-RISC total score, the cognitive composite, total number of self-reported concussions, and contact sport exposure at the professional level (i.e., years and games played professionally). We controlled for age and depressive symptoms in several analyses due to the potentially confounding impact of these variables on relationships between perceived (self-reported) cognitive decline and other variables of interest (17, 18, 21). Age was not included as a covariate in the correlations using the cognitive composite because the individual scores that comprise the cognitive composite were each age-adjusted based on normative data.

Next, three simultaneous multiple regression models were conducted. First, the IQCODE-Self was regressed on the following predictors: age, self-reported concussion history, contact sport exposure at the professional level (years played professionally), DASS depression, resilience (the CD-RISC), the cognitive composite, alcohol use (the AUDIT), sleepiness (the ESS), and the BPI pain severity rating. In the second and third regression models, the cognitive composite score and memory composite score were regressed on years of education, estimated intelligence (the ToPF), self-reported concussion history, contact sport exposure at the professional level (i.e., years played professionally), DASS depression, resilience (the CD-RISC), perceived cognitive decline (the IQCODE-Self), alcohol use (the AUDIT), sleepiness (the ESS), and BPI pain severity.

In each regression model, normality and homoscedasticity were assessed through a visual inspection of the plot of standardized predicted values against standardized residuals. Outliers and influential cases were measured by examining standardized residuals (with values ±1.96 reflecting unusual cases) and Cook's distance (with > 1 defining problematic influential cases). Multicollinearity was defined as intercorrelations of > 0.8 among predictors, variance inflation factor (VIF) values > 10, or tolerance values < 0.1. Model cross-validation was assessed by examining Adjusted *R*^2^ in addition to standard *R*^2^.

The False Discovery Rate was used to control for Type I error in correlational analyses associated with the hypotheses, with alpha set at *p* < 0.05 (two-tailed tests). The False Discovery Rate predicts and controls individual false positive results, while simultaneously maintaining a high level of statistical power relative to familywise error rate methods such as the Bonferroni correction (55). The False Discovery Rate was computed using an online calculator. Data were analyzed with SPSS version 27.0.

## Results

### Demographic Characteristics and Exposure History

The median age of the sample of 133 retired NRL players was 55.0 (M = 53.1, SD = 13.9; IQR = 41.0–64.0, range = 30–89) and their median years of education completed was 12.0 (M = 11.9, SD = 2.6; 10.0–13.0, range = 7–18). Approximately 72.2% of retired players were married, 16.5% were divorced, 7.5% were single, 2.3% were cohabitating, and 1.5% were separated. Approximately 75.9% of the retired players were employed full time, 3.0% were employed part time, 19.5% were retired, and 1.5% were unemployed. The retired players reported a median of 15.0 total lifetime concussions (M = 28.0, SD = 36.6). Exposure history variables are presented in Table 1.

**Table 1:** Self-reported exposure history.

	***n***	**Mean**	**SD**	**Md**	**IQR**	**Range**
Self-reported concussion history	133	28.0	36.6	15.0	6.0–29.5	0.0–200.0
Number of concussions with LOC	133	4.2	5.9	3.0	1.0–5–5	0.0–50.0
Years played professionally	133	8.1	4.8	9.0	3.5–11.5	1.0–20.0
Games played professionally	130	123.0	101.9	96.5	21.8–213.5	1.0–357.0
Total (lifetime) years played	133	23.9	5.5	25.0	21.0–27.0	7.0–37.0

*LOC, loss of consciousness; SD, standard deviation; Md, median; IQR, interquartile range*.

### Health History, Self-Report, and Cognitive Measures

Descriptive statistics for the self-report measures are provided in Table 2. Participants' mean IQCODE-Self score was 3.2 (SD = 0.5; Range = 1.3–5.0). Of the 133 retired athletes, 10 (7.5%) and 38 (28.6%) scored above the 3.88 and 3.38 cutoffs on the IQCODE-Self, respectively. Their longstanding intellectual ability, as estimated from a reading test (ToPF), was estimated to be in the average range (M = 99.2, SD = 10.0), and their overall performance on neuropsychological testing, as measured by the cognitive composite score, was also in the average range (M = 48.2, SD = 6.9; Table 2). A lifetime history of depression was reported by 29.3% of the sample (Table 3). Regarding current symptoms of depression, 30.0% of the retired players reported at least mild depression on the DASS (see Table 3). The breakdown of participants by DASS depression classification ranges was as follows: Broadly Normal = 70.0%, Mild = 15.0%, Moderate = 10.5%, and Severe/Extremely Severe = 4.5% (Table 3).

**Table 2:** Self-report and cognitive measures.

	***n***	**Mean**	**SD**	**Md**	**IQR**	**Range**
Cognitive decline (IQCODE-Self)	133	3.2	0.5	3.1	3.0–3.4	1.3–5.0
Cognitive composite score (T score)	133	48.2	6.9	49.2	44.3–52.4	25.3–64.2
Memory composite score (T score)	132	41.6	8.4	42.5	36.1–46.9	21.8–59.3
ToPF standard score	133	99.2	10.0	98.0	93.0–107.0	70.0–122.0
Depression (DASS)	133	6.8	8.0	4.0	0.0–10.0	0.0–42.0
Anxiety (DASS)	133	4.3	5.5	2.0	0.0–6.0	0.0–32.0
Stress (DASS)	133	10.2	9.2	8.0	2.0–16.0	0.0–42.0
Resilience (CD-RISC)	132	74.6	14.7	77.0	67.0–85.0	38.0–100.0
Alcohol use (AUDIT)	133	7.4	5.0	6.0	10.0–13.0	7.0–18.0
Daytime sleepiness (ESS)	133	7.2	5.0	6.0	3.5–10.5	0.0–24.0
Pain severity (BPI)	132	2.1	2.2	1.9	0.0–3.8	0.0–7.3
Pain interference (BPI)	132	1.8	2.3	0.6	0.0–3.5	0.0–8.4

*AUDIT, Alcohol Use Disorders Identification Test; BPI, Brief Pain Inventory; CD-RISC, Connor Davison Resilience Scale; DASS, Depression, Anxiety, Stress Scales; ESS, Epworth Sleepiness Scale; IQCODE, Informant Questionnaire on Cognitive Decline in the Elderly; ToPF, Test of Premorbid Functioning. DASS, Depression Normal range = 0–9; IQCODE-Self cutoff values for cognitive impairment, 3.88 (conservative) and 3.38 (liberal)*.

**Table 3:** Past and present health problems.

**Current or recent…**		**Lifetime history of…**	
Cognitive decline (IQCODE-Self, cutoff = 3.88)	10 (7.5%)	Sleep apnea	8 (6.0%)
Cognitive decline (IQCODE-Self, cutoff = 3.38)	38 (28.6%)	Epilepsy	2 (1.5%)
DASS depression normal (0–9)	93 (70.0%)	Stroke	5 (3.8%)
DASS depression mild (10–13)	20 (15.0%)	*Family* history of dementia	43 (32.3%)
DASS depression moderate (14–20)	14 (10.5%)	Hypertension	19 (14.3%)
DASS depression severe (21+)	6 (4.5%)	High cholesterol	12 (9.0%)
AUDIT low-risk	75 (56.4%)	Myocardial infarction	3 (2.3%)
AUDIT risky or hazardous level	49 (36.8%)	Other heart condition	6 (4.5%)
AUDIT high-risk or harmful level	6 (4.5%)	Peripheral vascular disease	4 (3.0%)
AUDIT high-risk (likely dependence)	3 (2.3%)	COPD	3 (2.3%)
Recent cannabis use (past 6 months)	7 (5.3%)	Diabetes	8 (6.0%)
Recent illicit drug use (past 6 months)	17 (12.8%)	Thyroid disease	2 (1.5%)
**Lifetime history of…**		Liver disease	4 (3.0%)
Depression	39 (29.3%)	Renal insufficiency	2 (1.5%)
Psychiatric history	27 (20.3%)	Peptic ulcer	7 (5.3%)
Lifetime illicit drug use	37 (27.8%)	Cancer	12 (9.0%)
Headache	33 (24.8%)	Arthritis	69 (51.9%)
Migraine	14 (10.5%)		

*AUDIT, Alcohol Use Disorders Identification Test; COPD, Chronic obstructive pulmonary disease; DASS, Depression, Anxiety, Stress Scales; IQCODE, Informant Questionnaire on Cognitive Decline in the Elderly*.

An exploratory bivariate correlation matrix with self-report measures, the cognitive composite score, demographic variables, and exposure variables is presented in Table 4. Most correlations were small and non-significant. As expected, measures of current psychological distress (i.e., depression, anxiety, and stress) were strongly positively intercorrelated (*r*s = 0.54–0.58). Resilience had a strong negative correlation with depression (*r* = −0.54) and medium negative correlations with anxiety (*r* = −0.33) and stress (*r* = −0.31). There were small positive correlations between life interference due to pain and the psychological distress measures (depression: *r* = 0.21, anxiety: *r* = 0.20, and stress: *r* = 0.24).

**Table 4:** Bivariate correlation matrix.

	**1**	**2**	**3**	**4**	**5**	**6**	**7**	**8**	**9**	**10**	**11**	**12**	**13**	**14**	**15**
1. Perceived cognitive decline (IQCODE-self)	–	–	–	–	–	–	–	–	–	–	–	–	–	–	
2. Cognitive functioning (composite score)	−0.16	–	–	–	–	–	–	–	–	–	–	–	–	–	
3. Depression (DASS)	0.33^*^	−0.15	–	–	–	–	–	–	–	–	–	–	–	–	
4. Anxiety (DASS)	0.20^*^	−0.10	0.54^*^	–	–	–	–	–	–	–	–	–	–	–	
5. Stress (DASS)	0.12	−0.03	0.57^*^	0.58^*^	–	–	–	–	–	–	–	–	–	–	
6. Resilience (CD-RISC)	−0.25^*^	0.07	−0.54^*^	−0.33^*^	−0.31^*^	–	–	–	–	–	–	–	–	–	
7. Education (years)	−0.08	0.34^*^	0.04	0.05	0.17	−0.05	–	–	–	–	–	–	–	–	
8. Estimated intelligence (ToPF)	−0.02	0.46^*^	−0.03	0.10	0.09	0.06	0.48^*^	–	–	–	–	–	–	–	
9. Pain severity (BPI)	0.14	0.04	0.15	0.16	0.21^*^	0.004	−0.03	0.17	–	–	–	–	–	–	
10. Pain interference (BPI)	0.18^*^	0.05	0.21^*^	0.20^*^	0.24^*^	−0.04	0.04	0.19^*^	0.93^*^	–	–	–	–	–	
11. Daytime sleepiness (ESS)	0.11	0.06	−0.11	−0.03	−0.17^*^	0.11	0.04	0.15	−0.09	−0.06	–	–	–	–	
12. Alcohol use (AUDIT)	−0.08	0.15	−0.09	−0.07	−0.04	0.01	0.10	0.16	−0.13	−0.14	−0.04	–	–	–	
13. Games played professionally	−0.25^*^	−0.04	−0.19^*^	−0.19^*^	−0.10	0.13	0.16	−0.03	−0.07	−0.13	0.02	0.09	–	–	
14. Years played professionally	−0.27^*^	−0.03	−0.17	−0.12	−0.09	0.16	0.08	−0.04	0.01	−0.08	0.05	0.08	0.82^*^	–	
15. Total (lifetime) years played	−0.07	−0.08	0.01	0.08	0.02	0.01	−0.03	−0.04	0.08	0.06	−0.10	0.14	0.36^*^	0.26^*^	
16. Number of self-reported concussions	0.12	−0.001	0.18^*^	0.10	−0.28^*^	−0.04	0.14	0.09	0.09	0.07	−0.06	−0.003	−0.02	−0.03	0.003

*The p-values were not adjusted for the false discovery rate for this exploratory correlation matrix (*

* *p < 0.05). AUDIT, Alcohol Use Disorders Identification Test; BPI, Brief Pain Inventory; CD-RISC, Connor Davison Resilience Scale; DASS, Depression, Anxiety, Stress Scales; ESS, Epworth Sleepiness Scale; IQCODE-Self, Informant Questionnaire on Cognitive Decline in the Elderly, self-report form; ToPF, Test of Premorbid Functioning*.

### Bivariate Predictors of, and Correlates With, Cognitive Decline

Hypothesis-driven correlations are presented in Table 5. The IQCODE-Self was positively correlated with depression, indicating that higher levels of perceived (self-reported) cognitive decline are associated with higher levels of depressive symptomatology; this relationship was independent of age. Perceived cognitive decline was negatively correlated with resilience, indicating that higher levels of perceived cognitive decline are associated with less resilience. However, the association between perceived cognitive decline and resilience was not significant when covarying for current depressive symptoms (but it remained significant when covarying for age). Contrary to our hypothesis, perceived cognitive decline was negatively correlated with professional rugby league exposure, suggesting that higher levels of perceived cognitive decline were associated with *fewer years* of professional sport exposure. Finally, perceived cognitive decline was not significantly associated with objective cognitive performance or lifetime history of concussions.

**Table 5:** Spearman bivariate and partial correlations between IQCODE-self and variables of interest.

	**IQCODE-Self**			
	**Bivariate**	**Partial Covariates: age, depression**	**Partial Covariate: age**	**Partial Covariate: depression**
Depression (DASS)	*r =* 0.33, *p* < 0.001	–	*r =* 0.35, *p* < 0.001	–
Resilience (CD-RISC)	*r =* −0.25, *p* = 0.02	*r =* −0.08, *p* = 0.37	*r =* −0.25, *p* = 0.02	*r =* −0.08, *p* = 0.37
Cognitive composite	*r =* −0.16, *p* = 0.11	–	–	*r =* −0.12, *p* = 0.21
Number of self-reported concussions	*r =* 0.12, *p* = 0.21	*r =* 0.14, *p* = 0.17	*r* = 0.19, *p* = 0.06	*r* = 0.07, *p* = 0.44
Years played professionally	*r =* −0.27, *p* = 0.01	*r =* −0.19, *p* = 0.07	*r =* −0.23, *p* = 0.02	*r =* −0.23, *p* = 0.02

*CD-RISC, Connor Davison Resilience Scale; DASS, Depression, Anxiety, Stress Scales; IQCODE-Self, Informant Questionnaire on Cognitive Decline in the Elderly, self-report form; The Spearman correlation between years played professionally and games played professionally is r = 0.82 (p < 0.001). The p-values have been corrected for the false discovery rate*.

### Multivariable Prediction Models

For the three multiple regression models, all Cook's distance values were < 0.45, and ~5% of cases had standardized residuals of ±1.96, which is to be expected in a normal distribution. These results suggested that the model was not unduly influenced by individual cases. Moreover, no intercorrelations among predictors exceeded 0.8, VIF values were all < 1.7, and tolerance values were all >0.62, suggesting that multicollinearity was not an issue. An inspection of the plot of standardized predicted values against standardized residuals suggested non-normality and heteroscedasticity. However, results from bootstrapped parameters (1,000 samples), which do not require these assumptions, did not differ from the original results.

Regression model parameters are presented in Tables 6–8. The overall IQCODE-Self model was significant (*p* = 0.004), with 18% of the variance in perceived cognitive decline accounted for by the set of predictors (Adjusted *R*^2^ = 0.11). The beta value for depression was larger than beta values for all other predictors, and it was the only statistically significant predictor of perceived cognitive decline, β = 0.29, *t* = 2.82, *p* < 0.01. The regression models that used the cognitive composite score (*p* < 0.001; *R*^2^ = 0.25, Adjusted *R*^2^ = 0.19) and memory composite score (*p* = 0.002; *R*^2^ = 0.20, Adjusted *R*^2^ = 0.14) as the dependent variable were also significant. In both models, the only significant predictor of the cognitive functioning was the estimated intelligence (ToPF) variable (cognitive composite, *p* < 0.001; memory composite, *p* = 0.001). All other predictors, including perceived cognitive decline, were not significant.

**Table 6:** Simultaneous multiple regression model with perceived cognitive decline (IQCODE-self) as the outcome.

	**B**	**SE B**	**β**	***t*** **(***p***)**	**Bootstrapped** **95% CI (lower, upper)** **for B**
Constant	2.88	0.60			
Age	0.01	0.003	0.17	1.76 (0.08)	−0.001, 0.01
Number of self-reported concussions	0.002	0.001	0.11	1.32 (0.19)	−0.001, 0.01
Years played professionally	−0.004	0.01	−0.04	−0.46 (0.65)	−0.02, 0.01
Depression (DASS)	0.02	0.01	0.29	2.82 (0.01)	0.002, 0.03
Resilience (CD-RISC)	−0.002	0.003	−0.06	−0.58 (0.56)	−0.01, 0.01
Cognitive composite score	−0.003	0.01	−0.04	−0.38 (0.70)	−0.02, 0.01
Alcohol use (AUDIT)	0.003	0.01	0.03	0.31 (0.76)	−0.02, 0.02
Daytime sleepiness (ESS)	0.02	0.01	0.16	1.89 (0.07)	−0.002, 0.04
Pain severity (BPI)	0.02	0.02	0.09	1.00 (0.32)	−0.03, 0.07

*R^2^ = 0.18, F_(9, 122)_ = 2.89, p = 0.004; Adjusted R^2^ = 0.11. AUDIT, Alcohol Use Disorders Identification Test; BPI, Brief Pain Inventory; CD-RISC, Connor Davison Resilience Scale; DASS, Depression, Anxiety, Stress Scales; ESS, Epworth Sleepiness Scale; IQCODE-Self, Informant Questionnaire on Cognitive Decline in the Elderly, self-report form*.

**Table 7:** Simultaneous multiple regression model with the cognitive composite score as the outcome.

	**B**	**SE B**	**β**	***t*** **(***p***)**	**Bootstrapped** **95% CI (lower, upper)** **for B**
Constant	26.59	7.91			
Education	0.27	0.25	0.10	1.08 (0.28)	−0.16, 0.67
Estimated intelligence (ToPF)	0.26	0.07	0.37	3.90 (<0.001)	0.14, 0.38
Number of self-reported concussions	−0.03	0.02	−0.14	−1.74 (0.09)	−0.06, 0.02
Years played professionally	−0.13	0.10	−0.10	−1.27 (0.21)	−0.33, 0.06
Depression (DASS)	−0.02	0.09	−0.03	−0.27 (0.79)	−0.18, 0.10
Resilience (CD-RISC)	0.01	0.04	0.01	0.10 (0.92)	−0.09, 0.09
Perceived cognitive decline (IQ-Code-Self)	−1.16	1.16	−0.09	−1.01 (0.32)	−4.09, 1.52
Alcohol use (AUDIT)	0.13	0.12	0.10	1.16 (0.25)	−0.10, 0.37
Daytime sleepiness (ESS)	−0.08	0.12	−0.06	−0.70 (0.48)	−0.34, 0.16
Pain severity (BPI)	−0.05	0.26	−0.02	−0.20 (0.84)	−0.58, 0.49

*R^2^ = 0.25, F_(10, 121)_ = 4.07, p < 0.001; Adjusted R^2^ = 0.19. AUDIT, Alcohol Use Disorders Identification Test; BPI, Brief Pain Inventory; CD-RISC, Connor Davison Resilience Scale; DASS, Depression, Anxiety, Stress Scales; IQCODE-Self, Informant Questionnaire on Cognitive Decline in the Elderly, self-report form; ToPF, Test of Premorbid Functioning*.

**Table 8:** Simultaneous multiple regression model with the Memory Composite Score as the outcome.

	**B**	**SE B**	**β**	***t*** **(***p***)**	**Bootstrapped** **95% CI (lower, upper)** **for B**
Constant	13.52	10.01	–	–	–
Education	0.36	0.31	0.11	1.14 (0.26)	−0.33, 0.96
Estimated intelligence (ToPF)	0.28	0.08	0.34	3.42 (0.001)	0.12, 0.46
Number of self-reported concussions	−0.01	0.02	−0.06	−0.66 (0.51)	−0.05, 0.35
Years played professionally	−0.09	0.13	−0.06	−0.67 (0.50)	−0.30, 0.14
Depression (DASS)	0.02	0.11	0.02	0.21 (0.83)	−0.21, 0.23
Resilience (CD-RISC)	0.06	0.06	0.10	1.02 (0.31)	−0.07, 0.17
Perceived cognitive decline (IQ-Code-Self)	−1.39	1.55	−0.08	−0.90 (0.37)	−4.78, 1.43
Alcohol use (AUDIT)	0.05	0.15	0.03	0.31 (0.76)	−0.20, 0.30
Daytime sleepiness (ESS)	−0.16	0.15	−0.09	−1.09 (0.28)	−0.42, 0.13
Pain severity (BPI)	−0.55	0.33	−0.15	−1.68 (0.10)	−1.30, 0.14

*R^2^ = 0.20, F_(10, 120)_ = 3.07, p = 0.002; Adjusted R^2^ = 0.14. AUDIT, Alcohol Use Disorders Identification Test; BPI, Brief Pain Inventory; CD-RISC, Connor Davison Resilience Scale; DASS, Depression, Anxiety, Stress Scales; IQCODE-Self, Informant Questionnaire on Cognitive Decline in the Elderly, self-report form; ToPF, Test of Premorbid Functioning*.

## Discussion

This is the first large study of subjectively experienced cognitive decline in retired elite level rugby league players. The proportion of the sample endorsing perceived (self-reported) cognitive decline on the IQCODE-Self varied depending on the cutoff used, with 7.5% scoring above a conservative value of 3.88 and 28.6% scoring above a liberal value of 3.38. Both of these estimates are lower than the value reported in a large survey study of retired NFL players, in which 1,502/3,758 (40.0%) of the sample endorsed daily cognitive problems (5). However, perceived cognition in that study was assessed with the Quality of Life in Neurological Disorders Item Bank (56), which measures the frequency of cognitive problems over the past 7 days, rather than the IQCODE-Self, which measures perceived cognitive decline over the last 10 years. Consequently, differences in cognitive symptom reporting may be explained, at least in part, by variation in samples and measurement of subjective cognition across studies.

Consistent with our hypotheses, perceived cognitive decline was positively correlated with depression, even when controlling for age, and no relationship was present between subjectively-experienced cognitive decline and objectively-measured cognitive functioning on neuropsychological testing. Moreover, depression was the only significant predictor of perceived cognitive decline in a multiple regression model. Partially consistent with the hypothesis, there was a negative correlation between perceived cognitive decline and resilience that became non-significant when controlling for depression. Contrary to our hypotheses, perceived cognitive decline was negatively associated with elite level rugby league exposure (i.e., years played professionally) and no relationship was observed between perceived cognitive decline and self-reported lifetime history of concussions. There was also no association between perceived cognitive decline and lifetime exposure to this collision sport. Finally, estimated intelligence was the only significant predictor of overall cognitive and memory performance in two multiple regression models.

### Perceived Cognitive Decline, Depression, and Objective Cognition

Our primary finding that depression was the most robust correlate and predictor of perceived cognitive decline is consistent with a large evidence base in the general population (17, 21). It is also consistent with empirical work in retired NFL players suggesting an association between cognitive complaints and depressive symptoms (5, 32). In fact, in the general population, depressive symptoms often explain observed relationships between subjective cognitive complaints and (a) TBI history (23) and (b) objective cognitive performance (17, 21). The robust relationship between cognitive concerns and depressive symptoms suggests that perceived cognitive decline and low mood “travel together” in some retired elite level rugby league players. Symptoms of depression and psychological distress can be successfully treated with evidence-based interventions such as cognitive behavioral therapy and/or antidepressant medications (57, 58), but some elite male athletes may be less likely to readily endorse mental health symptoms due to hypermasculinity and concerns about stigma (59–61). Consequently, when cognitive problems are reported, clinicians working with these men are encouraged to also carefully assess for depressive symptoms and other mental health concerns and then to provide evidence-based treatments as indicated.

### Additional Correlates of Perceived Cognitive Decline

In the current study, perceived (self-reported) cognitive decline was unrelated to the number of self-reported concussions. Several previous studies, comprised of former high school and college football players, as well as retired NFL players, have reported positive correlations between the number of prior concussions and later subjective cognitive complaints (4, 6, 7). Additionally, in a sample of 111 current and retired professional rugby union players, those who reported a history of three or more Grade 2 or 3 concussions (62) had greater subjective memory complaints than those who reported none (Cohen's *d* = −0.62) (63). Overall, it is not clear why we did not replicate past work suggesting a relationship between concussion history and perceived cognitive difficulties in former American football players. There are important differences in American football and rugby league such as the presence or absence of helmets and pads, the biomechanics of tackling, and possibly the rate and biomechanics of repetitive neurotrauma. The impact of these differences on clinical variables such as perceived cognitive decline is not well-understood and is beyond the scope of the present study. Another important factor is differences in participant recruitment and methodology across studies. Our sample of retired elite level rugby league players is unique compared to prior research in former rugby players, including some of our recruitment strategies (e.g., alumni networks). For example, Thornton et al. (63) recruited both current and retired Rugby Union players, while our sample was comprised entirely of retired rugby league players. Additionally, our retired athletes reported a high number of prior concussions (Mdn = 15; IQR = 6–29.5) compared to a historical survey of retired NFL players (6) (39.2% of the sample: 0 concussions, 36.8%: 1–2 concussions, 24%: 3+ concussions), which might reflect greater awareness of concussion now compared to 20 years ago. That said, it is unclear how this high number of self-reported injuries in our sample might impact the association between prior concussions and perceived cognitive decline.

We also measured perceived cognitive decline over the past 10 years using the IQCODE-Self, and the other cited studies used measures specific to current cognitive complaints. The IQCODE-Self requires the former athlete to reflect on his functioning over the past 10 years and determine whether he perceives a decline in memory, comprehension, decision making, reasoning, or instrumental activities of daily living. This is a fundamentally different task compared to assessing whether or not he believes that he is having current cognitive symptoms. Therefore, differences in culture (Australia compared to the U.S.), professional status (current vs. retired players), and measurement of subjective cognition may partially explain why our results differed from previous research.

In the current study, perceived cognitive decline was *negatively* correlated with elite level rugby league exposure (i.e., greater exposure to elite-level play was associated with less perceived cognitive decline), and there was no association between perceived cognitive decline and lifetime exposure to rugby league. Some researchers have reported that greater duration of contact/collision sport participation (primarily American football) is associated with probable REM sleep behavior disorder (64), white matter rarefaction and neurofibrillary tangles (65), cognitive decline reported by a collateral source (but not behavioral/mood symptoms) (66), self-reported cognition-related quality of life, (67) and extent of chronic traumatic encephalopathy pathology (68, 69). In contrast, Fields et al. (70) measured cognitive performance and mental health symptoms in 35 retired American football players and found no relationship between years played professionally and either objective cognitive performance or depressive symptoms. Overall, it is not entirely clear why our study results differ from some of the prior literature. Although speculative, it is possible that some current study participants with shorter playing careers were forced to retire due to injury, and qualitative case studies of forced retirement in elite athletes have found it to be associated with a sense of loss, a struggle to find meaning, and depressive symptoms (71, 72). In other words, one possible interpretation of the association between longer elite level rugby league careers and less perceived cognitive decline in the current study is that those athletes who play for more years are able to do so, in part, because they experience fewer serious sport-related injuries, while those who retire earlier face negative mental health consequences of doing so. It is also possible that we did not find the predicted positive association between rugby league exposure and perceived cognitive decline because we sampled all former players, regardless of their current symptomatology, whereas some prior studies only recruited players who were symptomatic at the time of the assessment (65, 66).

Our analyses revealed a negative relationship between perceived cognitive decline and resilience, which became non-significant when depression was covaried. Resilience is negatively correlated with mental health symptoms in current athletes (73–76). Moreover, Laird et al. (77) reported a negative correlation of *r* = −0.43 between resilience and subjective memory complaints in 288 older adults with major depressive disorder. Our results suggest that resilience shares some variance with perceived cognitive decline, but that depressive symptoms are a better overall indicator of subjective cognitive concerns.

### Limitations

The current study is limited in several ways. First and foremost, we cannot say that our sample is representative of all retired elite rugby league players because it represents those who volunteered to participate in this elaborate study relating to brain health. Some may have chosen to do so because they felt symptomatic and concerned about their functioning. It is possible that some former athletes who are feeling healthy and are leading active, productive lives chose not participate. In contrast, it is also possible that some former players who are cognitively impaired or who have neurological diseases were less likely, or not able, to participate. With that said, we did have three former players in the study with dementia severe enough that they could not meaningfully complete the self-report measures, and, consequently, they were not included in our analyses. Thus, overall, our sample might not fully represent either end of the cognitive health spectrum, from those who are the healthiest and highest functioning, to those who are the most neurologically compromised.

Second, the study sample was comprised of exclusively retired male rugby league players, so external validity is limited with respect to other populations. Third, the study design is cross-sectional and, therefore, we are unable to infer causality. Fourth, the memory composite T scores were lower than expected (M = 41.6, SD = 8.4), suggesting that there may be memory problems in a subset of the sample. However, we did not have a control group of retired non-contact athletes for comparison, so we interpret these normative scores with caution. Moreover, the focus of the current study is on perceived rather than objective cognitive decline, so we did not investigate cognitive or memory performance further.

Fifth, concussion history and contact sport exposure were measured retrospectively, via self-report, and this method is vulnerable to recall failures and biases. Finally, the construct of perceived cognitive decline itself is also subject to memory failures (17). That is, if a person's memory has truly declined to the point of being impaired, asking that person to recall and report on their memory and cognitive functioning over the past 10 years may not be an accurate indicator of their actual abilities.

### Conclusions

Subjective cognitive concerns are of interest to the scientific community at large (17, 19–21, 78, 79), and are present in retired elite athletes (5, 8). However, there is a dearth of evidence pertaining to cognitive and mental health correlates of perceived (self-reported) cognitive decline in retired contact or collision professional athletes (outside of American football). Overall, the current results suggest that perceived cognitive decline in former elite level rugby league players is associated with symptoms of depression and psychological distress, and not with objective cognitive impairments as measured by neuropsychological tests. Consequently, subjectively experienced cognitive difficulties might improve in tandem with mental health when former athletes are provided with evidence-based treatments for depression (e.g., cognitive behavioral therapy and/or selective serotonin reuptake inhibitors). Alternatively, some athletes may experience depressive symptoms in association with cognitive decline and neurodegenerative disease, and the current study cannot speak to causality in the relationship between perceived cognitive decline and mental health.

In the general population, subjective cognitive complaints are associated, longitudinally, with later cognitive decline and dementia (19–21). However, the *cross-sectional* association between subjective and objective cognition is small, with only about 1% of the variance shared (17, 18). Therefore, we cannot rule out the possibility that perceived cognitive decline might be a precursor to later objective cognitive decline and dementia in some retired athletes, so individuals with subjective complaints should be monitored and followed over time from a cognitive perspective.

## Data Availability Statement

The statistical analyses and underlying data supporting the conclusions of this article will be made available by the authors, without undue reservation.

## Ethics Statement

The studies involving human participants were reviewed and approved by The University of Newcastle Human Ethics Committee. The participants provided their written informed consent to participate in this study.

## Author Contributions

RV assisted with the literature review, helped conceptualize the statistical analyses, conducted the statistical analyses, and wrote portions of the manuscript. GI conceptualized the study, helped conceptualize the statistical analyses, and wrote portions of the manuscript. DT helped conceptualize the statistical analyses. CL assisted with securing funding. AG is the overall principal investigator, secured funding for the larger parent study, directed that study, collected all of the data and managed the database, helped conceptualize this study, and wrote portions of the manuscript. All authors critically reviewed drafts of the manuscript and read and approved the last version of this manuscript.

## Funding

This study was funded in part by the NSW Sporting Injuries Committee, the Brain Foundation, Australia, and the Priority Research Centre for Stroke and Brain Injury. The authors have received funding from the National Football League for a program of research entitled The Spectrum of Concussion: Predictors of Clinical Recovery, Treatment and Rehabilitation, and Possible Long-Term Effects (PI: GI), although this study was not directly sponsored by that grant. Unrestricted philanthropic support was provided by the National Rugby League, ImPACT Applications, Inc., the Mooney-Reed Charitable Foundation, and the Spaulding Research Institute. The funders were not involved in the study design, collection, analysis, interpretation of data, the writing of this article or the decision to submit it for publication.

## Conflict of Interest

GI serves as a scientific advisor for NanoDx™, Sway Operations, LLC, and Highmark, Inc. He has a clinical and consulting practice in forensic neuropsychology, including expert testimony, involving individuals who have sustained mild TBIs including athletes. He has received research funding from several test publishing companies, including ImPACT Applications, Inc., CNS Vital Signs, and Psychological Assessment Resources PAR, Inc. He has received research funding as a principal investigator from the National Football League, and salary support as a collaborator from the Harvard Integrated Program to Protect and Improve the Health of National Football League Players Association Members. DT serves as a consultant for REACT Neuro, Inc. Christopher Levi, MBBS, serves as an honorary consultant neurologist to the National Rugby League NRL providing a pro bono second opinion to current players regarding concussion assessment and management for the player's club doctor. AG serves as a scientific advisor for hitIQ, Ltd. He has a clinical practice in neuropsychology involving individuals who have sustained sport-related concussion including current and former athletes. He has been a contracted concussion consultant to Rugby Australia since July 2016. He has received travel funding or been reimbursed by professional sporting bodies, and commercial organizations for discussing or presenting sport-related concussion research at meetings, scientific conferences, workshops, and symposiums. Previous grant funding includes the NSW Sporting Injuries Committee, the Brain Foundation Australia, an Australian-American Fulbright Commission Postdoctoral Award, a Hunter New England Local Health District, Research, Innovation and Partnerships Health Research & Translation Centre and Clinical Research Fellowship Scheme, and the Hunter Medical Research Institute HMRI, supported by Jennie Thomas, and the HMRI, supported by Anne Greaves. He is currently funded through an NHMRC Early Career Fellowship, and the University of Newcastle's Priority Research Centre for Stroke and Brain Injury. He has received research funding from the National Rugby League NRL for the Retired Players Brain Health research program. The remaining authors declare that the research was conducted in the absence of any commercial or financial relationships that could be construed as a potential conflict of interest.

## Publisher's Note

All claims expressed in this article are solely those of the authors and do not necessarily represent those of their affiliated organizations, or those of the publisher, the editors and the reviewers. Any product that may be evaluated in this article, or claim that may be made by its manufacturer, is not guaranteed or endorsed by the publisher.
